# The HDAC Inhibitor TSA Ameliorates a Zebrafish Model of Duchenne Muscular Dystrophy

**DOI:** 10.1371/currents.md.8273cf41db10e2d15dd3ab827cb4b027

**Published:** 2013-09-17

**Authors:** Nathan M. Johnson, Gist H. Farr, Lisa Maves

**Affiliations:** Center for Developmental Biology and Regenerative Medicine, Seattle Children's Research Institute, Seattle, Washington, USA; Center for Developmental Biology and Regenerative Medicine, Seattle Children's Research Institute, Seattle, Washington, USA; Center for Developmental Biology and Regenerative Medicine, Seattle Children's Research Institute, Seattle, Washington, USA; Department of Pediatrics, University of Washington, Seattle, Washington, USA

## Abstract

Zebrafish are an excellent model for Duchenne muscular dystrophy. In particular, zebrafish provide a system for rapid, easy, and low-cost screening of small molecules that can ameliorate muscle damage in dystrophic larvae. Here we identify an optimal anti-sense morpholino cocktail that robustly knocks down zebrafish Dystrophin (<i>dmd</i>-MO). We use two approaches, muscle birefringence and muscle actin expression, to quantify muscle damage and show that the <i>dmd</i>-MO dystrophic phenotype closely resembles the zebrafish <i>dmd</i> mutant phenotype. We then show that the histone deacetylase (HDAC) inhibitor TSA, which has been shown to ameliorate the <i>mdx</i> mouse Duchenne model, can rescue muscle fiber damage in both <i>dmd</i>-MO and <i>dmd</i> mutant larvae. Our study identifies optimal morpholino and phenotypic scoring approaches for dystrophic zebrafish, further enhancing the zebrafish <i>dmd</i> model for rapid and cost-effective small molecule screening.

## Introduction

Muscular dystrophies are genetic disorders characterized by progressive muscle degeneration and impaired muscle function. Many muscular dystrophies are caused by mutations in genes that encode components of the Dystrophin-glycoprotein complex 1 ^,^ 2, including Duchenne muscular dystrophy (DMD), which is caused by mutations in the Dystrophin (*DMD*) gene 3. Mechanisms that contribute to muscle degeneration in the muscular dystrophies include muscle membrane instability, disrupted calcium homeostasis, and oxidative stress 2. Gene-mediated and cell-mediated therapeutic strategies for DMD hold tremendous promise, but these approaches still face many obstacles 4 ^,^ 5 ^,^ 6. Therefore, many different pharmacological therapies are currently being pursued^5^^,^ 7 ^,^ 8.

The zebrafish, *Danio rerio*, offers several advantages as a model system for screening for chemical modifiers of the muscular dystrophy phenotype. First, zebrafish can be produced readily in large numbers and provide a rapid and cost-effective system for chemical screening 9. Second, zebrafish represent excellent models of human muscular dystrophies, in particular loss of Dystrophin to model DMD 10 ^,^ 11 ^,^ 12. Third, recent approaches have demonstrated pharmacological rescue of the zebrafish *dmd *mutant and other zebrafish myopathy models 13 ^,^ 14 ^,^ 15 ^,^ 16.

Even with these significant advantages, limitations in the zebrafish DMD model remain. With the zebrafish *dmd *mutant strain, about 25% of the embryos from a cross of heterozygote carriers show the recessive *dmd *muscle lesion phenotype 10 ^,^ 11 ^,^ 13. Therefore, when using zebrafish *dmd *clutches for chemical screening, phenotypic rescue can be assessed only on these 25% mutants while 75% of the clutches are phenotypically normal. Furthermore, to accurately assess rescue, the mutant individuals must be genotyped. Multiple previous studies have used antisense morpholino oligos to knock down zebrafish *dmd*, but these studies report achieving a dystrophic phenotype in only about 30% of morpholino-treated animals 10 ^,^ 13 ^,^ 17. We thus wanted to identify an improved zebrafish *dmd *morpholino knock-down approach for chemical screening and also for rapid use in other zebrafish mutant and transgenic backgrounds.

Here we develop a robust zebrafish *dmd *morpholino (*dmd*-MO) knock-down model that closely resembles the zebrafish *dmd *mutant phenotype and achieves almost 100% penetrance. We show that this *dmd*-MO model is useful for identifying small molecules that rescue the *dmd *phenotype by showing that the histone deacetylase (HDAC) inhibitor TSA, which has been shown to ameliorate the *mdx *mouse DMD model 18 ^,^ 19 ^,^ 20 ^,^ 21, can rescue muscle fiber damage similarly in both *dmd*-MO and *dmd *mutant larvae. Our study identifies optimal morpholino and phenotypic scoring approaches for dystrophic zebrafish, further enhancing the zebrafish *dmd *model for rapid and cost-effective small molecule screening.

## Materials and Methods

**Zebrafish husbandry**

All experiments involving live zebrafish (*Danio rerio*) were carried out in compliance with IACUC guidelines. Zebrafish were raised and staged as previously described 22. Time (hpf or dpf) refers to hours or days postfertilization at 28.5°C. The wild-type stock used was AB. The *Tg*(*acta1a*:*gfp*) line that labels skeletal muscle actin has been described 23. The *dmd^ta222a^* mutant line (also known as *sapje*) has been described and is a null allele 10 ^,^ 24. *dmd^ta222a ^*genotyping was performed as described 25.

**Morpholino injections**

Morpholino (MO) injections were performed as previously described 26 using a Narishige IM 300 Microinjector. MOs used were *dmd*-MO1, 5'-TTGAGTCCTTTAATCCTACAATTTT-3', and *dmd*-MO6, 5'-GCCATGACATAAGATCCAAGCCAAC-3' 11. MOs were initially tested individually at several doses injected in a volume of 1 nl into 1-cell stage embryos. For our strong *dmd*-MO cocktail, MOs were combined at the following working concentrations: *dmd*-MO1, 4 mg/ml; *dmd*-MO6, 7.5 mg/ml, and injected in a volume of 1 nl into 1-cell stage embryos. Non-injected sibling embryos served as controls.

**Wholemount immunocytochemistry**

Whole-embryo immunostaining was performed with the following primary antibodies: anti-Dystrophin, 1:100 (Sigma D8043); anti-GFP, 1:250 (Torrey Pines TP401). Secondary antibodies used were: goat anti-mouse AlexaFluor-568 and goat anti-rabbit AlexaFluor-488 (1:250, Life Technologies/Molecular Probes). Stainings were performed as previously described 27. To quantitate anti-Dystrophin staining (Fig. 1C), staining intensity per animal was assigned a value between 0 and 3, where 0 is no staining and 3 is the brightest control staining.

**Phalloidin staining**

For visualizing actin, phalloidin staining was performed as previously described 15 using Alexa-488 phalloidin (Life Technologies) on *dmd^ta222a ^*larvae and rhodamine-phalloidin (Cytoskeleton) on*Tg(acta1a:gfp) *larvae.

**Scoring and imaging muscle lesions**

For scoring numbers of animals with muscle lesions, whole embryos and larvae were either anaesthetized with tricaine 22 or fixed in 4% paraformaldehyde and viewed using an SZX16 Olympus stereomicroscope. Birefringence was imaged as previously described 28. Animals with gaps in *acta1a:gfp*, phalloidin, or birefringence patterns were scored as affected. For scoring numbers of myotomes with lesions per animal, larvae were mounted in 70% glycerol in PBS under a coverslip and imaged using a Leica TCS SP5 confocal microscope. 10 somites, approximately between the levels of somite 5 and 15, were imaged per animal. Somites with gaps in *acta1a:gfp *or phalloidin patterns were scored as affected. To image birefringence on the confocal, a single polarizing filter was placed between the filter turret and the condenser of the inverted microscope, and images were collected using the visible laser and brightfield illumination settings.

**TSA treatments**

A 30 µM stock solution of Trichostatin A (TSA, T8552, Sigma) was made in DMSO. Embryos were treated with 200 nM TSA in Embryo Medium (EM) 22 for three days, beginning at 24 hpf, and were fixed in 4% paraformaldehyde at 96 hpf for scoring muscle lesions. EM containing TSA or DMSO vehicle control was changed every 24 hours. Groups of 30 control or *dmd*-MO embryos were treated in 35 mm dishes containing 3 ml medium. Groups of approximately 80 embryos from *dmd^ta222a ^*heterozygote matings were treated in 60 mm dishes containing 9 ml medium.

**Statistical analyses**

Error bars in graphs report standard deviation, and significance was calculated using two-tailed, paired Student’s *t *tests.

## Results

**Identifying a robust zebrafish *dystrophin* morpholino knock down**

Multiple previous studies using morpholinos to knock down zebrafish *dystrophin *(*dmd*) reported achieving a dystrophic phenotype in about 30% of morpholino-treated animals 10 ^,^ 13 ^,^ 17. We wanted to identify an improved zebrafish *dmd *morpholino knock-down approach that would allow for robust and rapid *dmd *knock down for chemical screening. Our previous studies, and those of others, have shown that cocktails of non-overlapping morpholinos targeted against a single gene can provide more robust knock down and reduce non-specific morpholino toxicity than by using individual morpholinos 26 ^,^ 29 ^,^ 30 ^,^ 31. Therefore, we obtained two previously described, non-overlapping morpholinos designed to target near the translation start site of zebrafish *dmd*, *dmd*-MO1 and *dmd*-MO6 11 (nomenclature according to ZFIN, http://zfin.org). To identify a dose for each individual morpholino to use in a cocktail, we first determined whether there were doses of the individual morpholinos that would cause non-specific effects. We injected a series of doses of each morpholino and assessed whether the animals formed a swim bladder at 5 days post fertilization (dpf), a phenotype we have previously used to assess morpholino toxicity 26. We find that even a 10 ng dose of *dmd*-MO1 allows larvae to form swim bladders (Fig. 1A). With *dmd*-MO6, doses up to 7.5 ng allow for swim bladder formation (Fig. 1A). This swim bladder test thus identifies a maximum dose of 7.5 ng for *dmd*-MO6. We next asked whether doses of individual MOs could cause a dystrophic phenotype at 2 dpf. We assessed dystrophic muscle by injecting the *dmd *MOs into embryos carrying the skeletal muscle transgene *acta1a:gfp* 23, which reveals muscle lesions in *dmd *mutants 10 (this work, Fig. 3). We observe that, for *dmd*-MO1, increasing doses lead to increased penetrance, with a plateau at 4 ng. For *dmd*-MO6, increasing doses also lead to increased penetrance (Fig. 1B). We then tested whether doses of individual MOs could reduce Dystrophin expression at 2 dpf, as assessed with anti-Dystrophin staining in larvae. We observe that, for *dmd*-MO1, increasing doses lead to increasing reduction of Dystrophin, while for *dmd*-MO6, strong reduction of Dystrophin only occurs at 7.5 ng (Fig. 1C-H). Taking into account these three analyses, we settled on doses of 4 ng for *dmd*-MO1 and 7.5 ng for *dmd*-MO6 to combine for our *dmd*-MO cocktail. When we inject the *dmd*-MO cocktail, we find loss of anti-Dystrophin staining in all injected animals (Fig. 1C, 1I). Thus, our approach to determining individual MO doses identifies a cocktail that causes strong zebrafish Dystrophin knock down. Our subsequent *dmd*-MO experiments refer to this MO1 4 ng/MO6 7.5 ng combination.

**Identification of morpholino doses for zebrafish dmd knockdown.**
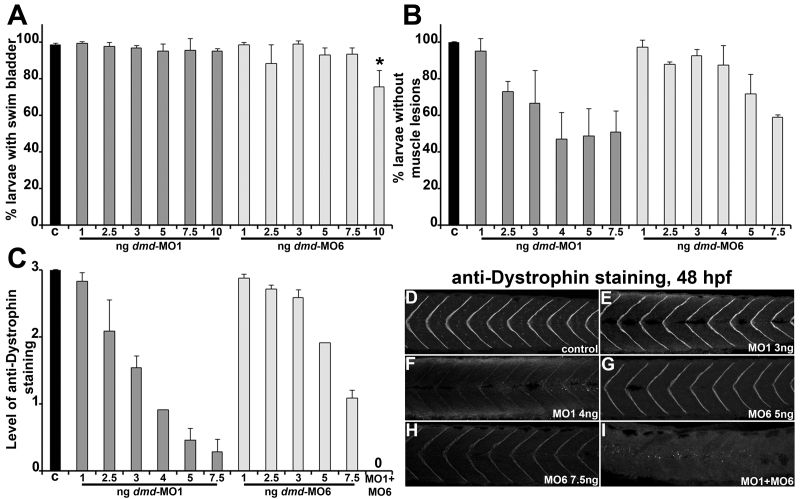
(A-C) Graphs of *dmd *morpholino (MO) effects. (A) Swim bladders were scored at 5 dpf. For each bar, n=3 with ≥25 larvae for each replicate. * P<0.04 versus control. (B) Muscle lesions were scored based on *acta1a:gfp *expression at 2 dpf. For each bar, n=3 with ≥30 larvae for each replicate. (C) Anti-Dystrophin staining was performed at 48 hpf. X-axes show ng of MO injected; c refers to controls. Anti-Dystrophin staining levels are arbitrary units. For each bar, n=2-3 with ≥12 larvae for each replicate. (D-I) Anti-Dystrophin staining in 48 hpf larvae. Lateral views of trunk somites show anterior to the left. Staining accumulates at myotome boundaries (Bassett et al., 2003; Guyon et al., 2003). In (C) and (I), MO1+MO6 refers to MO1 4 ng/MO6 7.5 ng combination.

**The *dmd*-MO muscle lesion phenotype strongly resembles that of *dmd* mutants**

To determine how well the *dmd*-MO cocktail phenocopies the *dmd *mutant, we took two approaches to analyzing muscle damage: muscle birefringence pattern and muscle actin labeling. Zebrafish muscle appears bright under polarized light, but zebrafish *dmd *mutants show disruptions in this birefringence due to muscle lesions caused by detachment of fibers from the myotendinous junctions 24 ^,^ 32 ^,^ 33. Similarly, muscle actin labeling with the transgene *acta1a:gfp *or with phalloidin reveals muscle lesions in dystrophic zebrafish 10 ^,^ 15. At 24 hpf, the muscle birefringence pattern is not yet developed, but *dmd*-MO and *dmd *mutant embryos show normal actin labeling (Fig. 2A-D). However, at 48 hpf and 96 hpf, both *dmd*-MO and *dmd *mutant larvae show strongly disrupted birefringence and actin labeling patterns (Fig. 2E-I). By 96 hpf, over 90% of *dmd*-MO larvae show disrupted birefringence and actin labeling patterns, similar to the 100% affected *dmd *mutants (Fig. 2E-H). Defects in actin labeling appear in a similar percentage of larvae as the disrupted birefringence (Fig. 2H-I). Thus, in *dmd*-MO animals, as in *dmd *mutants, muscle structure initially appears normal and then progressively worsens. These results show that our *dmd*-MO cocktail strongly phenocopies the *dmd *mutant muscle lesion phenotype.

**dmd-MO animals have a high penetrance of muscle lesions and resemble dmd mutants.**
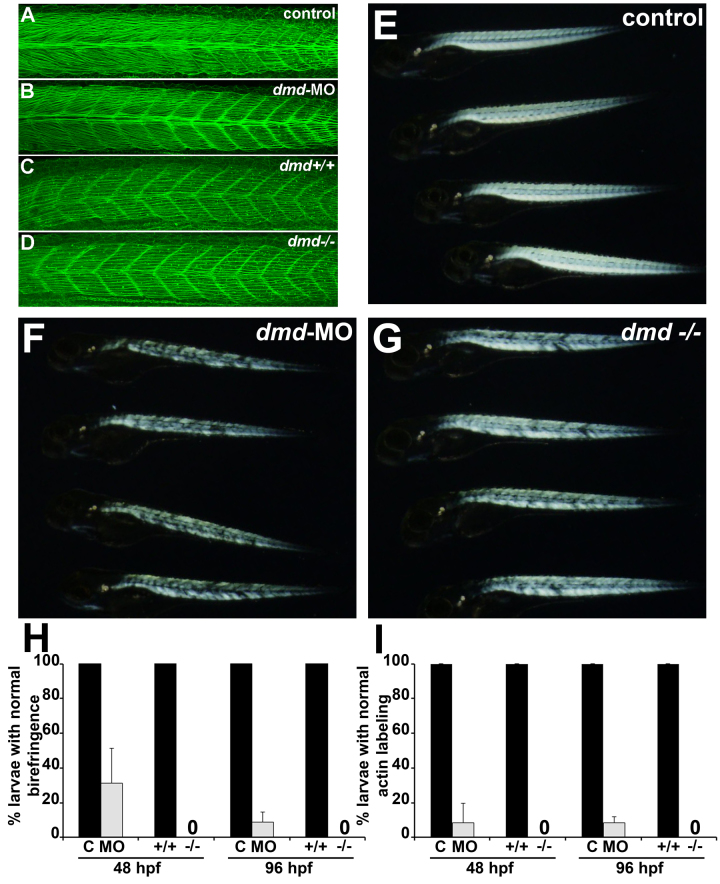
(A-D) Phalloidin staining in 24 hpf embryos. Lateral views of trunk somites show anterior to the left. (E-G) Birefringence images of 4 dpf larvae. Anterior to the left. (H-I) Quantification of larval birefringence and actin labeling patterns. For each bar, n=3-6 with ≥11 larvae for each replicate. For each condition, P<0.0006 relative to paired control sample. All larvae from *dmd* crosses were genotyped.

We next wanted to confirm that birefringence and actin labeling are similar reporters for the *dmd *muscle lesion phenotype. Using confocal microscopy to more closely examine muscle lesions, we find that there is a strong correlation among the birefringence, *acta1a:gfp*, and phalloidin patterns, and all three approaches reveal the same muscle lesions (Fig. 3). These results show that these three approaches are indeed similar reporters for *dmd *muscle lesions.

**Correlation among birefringence, acta1a:gfp, and phalloidin patterns.**
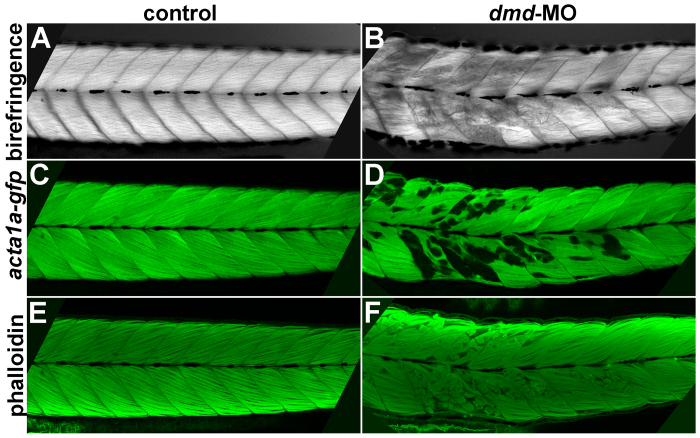
Confocal images of a single control (A,C,E) and *dmd*-MO (B,D,F) larva at 4 dpf. Phalloidin staining was imaged using the red channel but false-colored in green in E,F. Lateral views of trunk somites show anterior to the left. The birefringence, *acta1a-gfp*, and phalloidin muscle lesion patterns strongly correlate in all larvae that were examined for all three patterns (n=8). Abnormal birefringence also correlates with lesions visualized with phalloidin staining in *dmd-/-* larvae (n=13, not shown).

We noticed that not all muscle segments contained muscle lesions in *dmd*-MO larvae, consistent with the variability described in *dmd *mutants 10. To more quantitatively compare muscle damage in *dmd*-MO larvae with that in *dmd *mutants, we used confocal microscopy to score the percent myotomes per larva with muscle lesions, similar to the approach previously described 15. At 48 hpf and 96 hpf, both *dmd*-MO and *dmd *mutant larvae show a similar percentage of myotomes with lesions based on disrupted actin labeling (Fig. 4). Consistent with our per larva scoring above (Fig. 2), these results show that our *dmd*-MO cocktail strongly phenocopies the *dmd *mutant muscle lesion phenotype.

**dmd-MO animals have a similar percentage of affected myotomes as dmd mutants.**
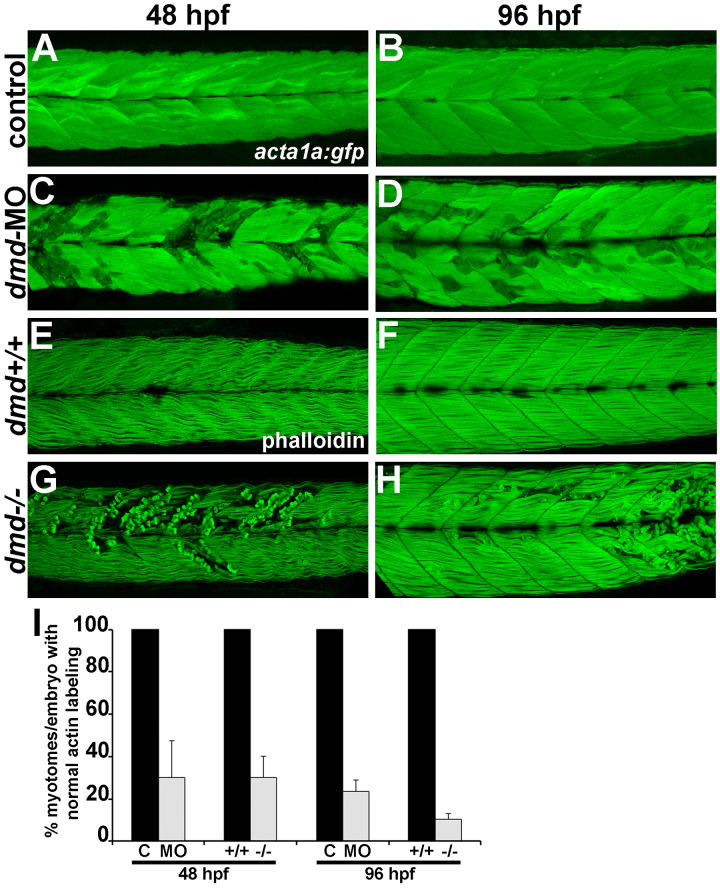
(A-D) *acta1a:gfp* expression. (E-H) phalloidin staining. Lateral views of trunk somites show anterior to the left. (I) Quantification of actin labeling patterns. For each bar at 48 hpf, n=3-6 with ≥ 11 larvae for each replicate. For each bar at 96 hpf, n=3 with ≥8 larvae for each replicate. For each condition, P<0.02 relative to paired control sample. All larvae from *dmd* crosses were genotyped.

**TSA rescues the muscle lesion phenotype of both *dmd*-MO larvae and *dmd* mutants**

Because our new *dmd*-MO cocktail induces a high frequency of animals with muscle lesions, we wanted to test whether we could use our *dmd*-MO to identify small molecules that could rescue the *dmd *phenotype. The histone deacetylase inhibitor TSA has been shown to ameliorate Duchenne muscular dystrophy in the mouse *mdx *model 18 ^,^ 19 ^,^ 20 ^,^ 21. We therefore asked whether TSA could rescue the muscle lesion phenotype in zebrafish *dmd*-MO larvae and* dmd *mutants. We began the TSA treatments at 24 hpf, which is prior to the appearance of the *dmd *phenotype and the stage when previous studies have initiated other chemical treatments that rescue the zebrafish *dmd *phenotype 13 ^,^ 14. Scoring both birefringence and actin labeling, we find that TSA treatment strongly rescues the muscle lesion phenotype in both *dmd*-MO larvae and *dmd *mutants (Fig. 5). Interestingly, the TSA rescue effect is similar whether scored per larva by birefringence pattern or per myotome with actin labeling (Fig. 5I-J). We confirmed that the TSA treatment did not restore Dystrophin expression in rescued *dmd*-MO or *dmd* mutant larvae (not shown). These results show that both the *dmd*-MO and *dmd *mutant zebrafish models are similarly and robustly rescued by TSA treatment and also show that assessing the muscle birefringence pattern in whole larvae, using a stereomicroscope, is a reliable approach to scoring the *dmd*-MO or *dmd *mutant phenotype following chemical treatment.

**Treatment with TSA rescues dmd-MO and dmd mutant muscle lesions.**
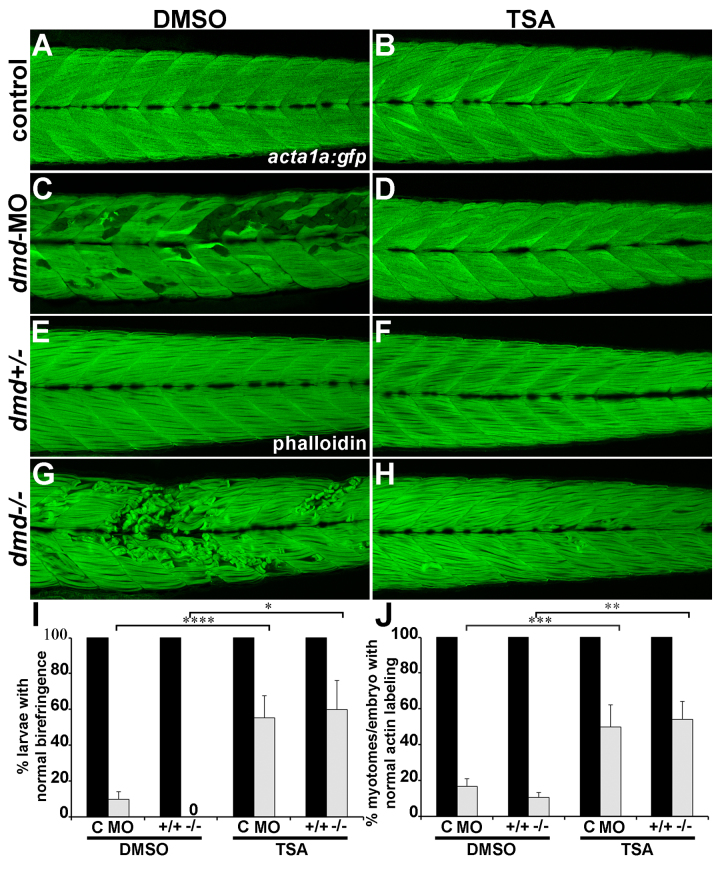
(A-D) *acta1a:gfp*expression. (E-H) phalloidin staining. Lateral views of trunk somites show anterior to the left. (I-J) Quantification of larval birefringence and actin labeling patterns. For control/*dmd*-MO treatments, for each bar, n=6 with ≥10 larvae for each replicate. For *dmd+/+/dmd-/-*treatments, for each bar, n=3 with ≥8 larvae for each replicate. * P<0.03. ** P<0.02. *** P<0.009. **** P<0.002. All larvae from *dmd *crosses were genotyped.

## Discussion

Our results identify a *dmd *morpholino cocktail that induces a high penetrance of muscle lesions and strongly resembles the zebrafish *dmd *mutant phenotype. We also show that the HDAC inhibitor TSA rescues both *dmd*-MO and *dmd *mutant muscle lesions. By comparing different approaches to scoring muscle lesions, our study confirms a previous report 13 that simple assessment of the muscle birefringence pattern in whole larvae, using a stereomicroscope, is a reliable approach to scoring the *dmd*-MO or *dmd *mutant phenotype following chemical treatment. Thus, our work identifies optimal morpholino and phenotypic scoring approaches for dystrophic zebrafish, further enhancing the zebrafish *dmd *model for rapid and cost-effective small molecule screening.

Our work provides additional support for using cocktails of non-overlapping morpholinos targeted against a single gene in order to provide more robust knock down and reduce non-specific morpholino toxicity than by using individual morpholinos 26 ^,^ 29 ^,^ 30 ^,^ 31. We previously reported using swim bladder scoring as an approach to identify working concentrations of individual morpholinos in zebrafish 26. Swim bladder scoring helped us to identify a working concentration for one of our *dmd *MOs (*dmd*-MO6, see Fig. 1A), but *dmd*-MO1 does not affect swim bladder formation, even at very high concentrations (Fig. 1A). Therefore, we used two additional approaches, *dmd *muscle phenotype and Dmd protein expression, to help determine the MO working concentrations. Based on this and our previous studies 26 ^,^ 31, we suggest identifying working concentrations of individual MOs that each have subtle effects on embryonic or larval phenotype for use in cocktails.

Several previous studies have demonstrated quantitative approaches to scoring dystrophic muscle lesions in zebrafish 13 ^,^ 14 ^,^ 15 ^,^ 33. For chemical screening, it is ideal to optimize the cost and speed of the screening approach while still allowing for reliable phenotypic scoring. We find that simple assessment of the muscle birefringence pattern in whole larvae, using a stereomicroscope, provides a comparable measurement of *dmd *phenotypic rescue by TSA treatment as the more quantitative, but time-consuming, approach of scoring myotome lesions using the confocal microscope. Also, in spite of having to inject embryos to generate *dmd*-MO larvae, we find that assaying *dmd*-MO larvae for rescue by TSA saves time and resources over using *dmd *mutant larvae, which require genotyping. There are, however, three potential disadvantages to using *dmd*-MO larvae. First, although we have not examined the *dmd*-MO phenotype past 5 dpf, the *dmd*-MO larvae likely are not useful for examining longer-term chemical rescue effects because the morpholinos will become diluted. Second, without using more quantitative measures of birefringence 14 ^,^ 33, it is possible we would not be able to distinguish phenotypic rescue by a drug that subtly improved muscle structure but still caused reduced birefringence. Third, even with the high penetrance of the *dmd*-MO phenotype, there are still approximately 20% or so larvae that appear not affected, whereas 0% of *dmd* mutants are not affected. This could be an important consideration for drug screening, where, if small numbers of fish were screened, the incomplete *dmd*-MO penetrance might cause false positive hits. Nevertheless, our work helps identify optimal morpholino and phenotypic scoring approaches for *dmd *drug screening. Candidate drugs that rescue *dmd*-MO larvae can be retested on *dmd *mutants.

The ability of TSA to ameliorate muscular dystrophy in the mouse *mdx *model may work through more than one mechanism. Initial studies of TSA and *mdx *rescue suggested that TSA acted through promoting upregulation of *follistatin *expression in satellite cells 18. A recent study, however, showed that fibroadipogenic progenitor cells mediate the ability of TSA to ameliorate muscular dystrophy in young *mdx *mice 21. Our demonstration that TSA can rescue the zebrafish *dmd *model now provides an additional model system for further mechanistic analysis of how TSA, and other small molecules, function to ameliorate dystrophic muscle.

## Correspondence

Lisa Maves. Email: lmaves@u.washington.edu

## Author Contributions

NMJ, GHF and LM conceived the experiments. NMJ and GHF performed the experiments. NMJ, GHF and LM analyzed the data. LM wrote the paper with contributions from NMJ and GHF.

## Competing Interest Statement

The authors have declared that no competing interests exist.
